# Omega-3 Polyunsaturated Fatty Acids Alleviate Traumatic Brain Injury by Regulating the Glymphatic Pathway in Mice

**DOI:** 10.3389/fneur.2020.00707

**Published:** 2020-07-17

**Authors:** Erwei Zhang, Xiangdong Wan, Lijun Yang, Dong Wang, Zeshang Chen, Yan Chen, Minghao Liu, Gengshen Zhang, Jianliang Wu, Haie Han, Zhenzeng Fan

**Affiliations:** ^1^Department of Neurosurgery, The Second Hospital of Hebei Medical University, Shijiazhuang, China; ^2^Department of Neurology, The Second Hospital of Hebei Medical University, Shijiazhuang, China

**Keywords:** omega-3 polyunsaturated fatty acid, traumatic brain injury, glymphatic system, aquaporin-4, blood-brain barrier, epilepsy

## Abstract

**Background:** The glymphatic pathway has been shown to be impaired in traumatic brain injury (TBI). Omega-3 polysaturated fatty acids (Omega-3, PUFAs) are involved in the clearance of amyloid-ß through the glymphatic system and this effect is Aquaporin-4 (AQP4) dependent. We hypothesize that Omega-3 PUFAs can alleviate neurological impairment in TBI by protecting the glymphatic pathway.

**Methods:** We pretreated mice with Omega-3 PUFAs rich fish oil and introduced TBI in the mice. Neurological functions were assessed through the modified neurological severity score (mNSS) system and Rota-rod test. Aß42 levels and radioisotope clearance were examined to determine the function of glymphatic system. AQP4 protein and mRNA expressions and its polarity were examined in fish oil treated TBI mice or control mice. Finally, the integrity of blood-brain barrier was determined by Evans blue extravasation and measurement of tight junction proteins (ZO-1 and Occludin) levels.

**Results:** TBI surgery induced significant neurological functional impairment, Omega-3 PUFAs attenuated TBI-induced neurological impairment, as evidenced by reduced mNSS, improved performance in the Rota-rod test. Furthermore, Omega-3 PUFAs improved glymphatic clearance after induction of TBI in mice, reduced Aß42 accumulation, partially restored the clearance of both ^3^H-mannitol and ^14^C-Inulin. Omega-3 PUFAs also suppressed AQP4 expression and partially prevented loss of AQP4 polarity in mice undergoing TBI. Finally, Omega-3 PUFAs protected mice from TBI induced blood-brain barrier disruption.

**Conclusion:** Omaga-3 PUFAs attenuate neurological function by partially restoring the AQP4 dependent glymphatic system in mice with TBI.

## Introduction

A recently identified system for clearance of waste substances in the brain, the glymphatic system eliminates harmful metabolites and soluble proteins from the central nervous system and mediates distribution of neuromodulators, growth factors, and other compounds throughout the brain through perivascular passages composed of astrocytes (1). Disruption of the glymphatic system has been shown to mediate chronic traumatic encephalopathy and brain trauma (2). This perivascular pathway exerts its function by facilitating the exchange of cerebrospinal fluid (CSF) and the interstitial fluid (ISF) (3). The Aquaporin 4 (AQP4) water channels are a key component of these perivascular tunnels that are mainly localized to the astrocytes in a polarized manner, with its accumulation found in the endfoot membrane of the astrocytes (4). AQP4 may be involved in Alzheimer's disease by facilitating clearance of neurotoxic amyloid ß (Aß) (5). Knockout of AQP4 in mice is associated with significant impairment of glymphatic function, leading to reduced clearance of solutes form the interstitial spaces, including Aß and mannitol (6). Perturbation of AQP4 expression or disruption in the polarity of the AQP4 protein leads to glymphatic dysfunction, which have been shown in several neurological diseases including stroke, Alzheimer's disease and traumatic brain injury (TBI) (7).

Epilepsy is closely related to the dysfunction of glymphatic system. Studies showed that potassium clearance was prolonged in hippocampal CA1 region on AQP4 gene knockout mice, which leading epileptic seizures by increasing neuronal excitability.

TBI, or sudden damage of the brain resulted from violent jolt or blow to the head, is a significant risk factor for neurodegenerative diseases including Parkinson's disease and Alzheimer's disease (8, 9). TBI is associated with various neurological dysfunctions, including impaired cognitive function and motor coordination such as poor performances in the Morris water maze test and wire hang test (10). Recently, multiple studies have explored the association between TBI and impairment in the glymphatic function and have shown that TBI leads to disruption in the waste clearance of the glymphatic system, resulting in accumulation of neurotoxic substances such as Amyloid ß (Aß) and Tau aggregations (11). Patients undergone severe TBI showed marked elevation of Aß42 levels in ventricular cerebrospinal fluid while plasma Aß42 did not change after injury (12). Deletion of the AQP4 gene in mice undergoing TBI stimulation further exacerbated the impaired clearance of interstitial solutes. Additionally, blood-brain barrier, a unique structure of the central nervous system that is important for brain homeostasis and substance exchange for proper function of the nervous system, has also been shown to be undermined by TBI (10). Fluid exchange and solute clearance of both the blood-brain barrier and glymphatic system are mediated by the AQP4 water channels (13). It is thus possible that restoration of the blood-brain barrier and glymphatic system or AQP4 protein function may prevent or alleviate TBI induced impairment of neurological functions.

Omega-3 polyunsaturated fatty acids (Omega-3 PUFAs) have been shown to have neuroprotective effects against neurodegenerative diseases and nutritional supplement of Omega-3 has been associated with a lower risk of Alzheimer's disease (14). In a mouse model of TBI induced by controlled cortical impact (CCI), nutritional supplementation of the mice with fish oil which is rich in omega-3 polyunsaturated fatty acid leads to improved recovery of neurological function (10). Another study also shows that treatment of mice prior to Aß injection protects against Aß induced neuronal toxicity by promoting Aß clearance through the glymphatic system and the effect is dependent on AQP4 (15). Previously, it has been shown that omega-3 polyunsaturated fatty acid promoted Aß clearance which was blocked by loss of AQP4 (15). Additionally, nutritional supplementation including omega-3 also reversed expression of AQP4 in rats undergoing mild TBI (16). We thus hypothesized that fish oil supplementation could alleviate TBI induced impairment in neurological functions through restoration of the glymphatic system and that AQP4 is involved in this process. This study will investigate the potential beneficial effects of Omega-3 in promoting waste clearance thorough the glymphatic system in the CCI induced TBI mouse model and explore the underlying molecular mechanism for this regulation.

## Materials and Methods

### TBI Mouse Model and Nutritional Supplementation

All experiments were approved by the institutional ethics committee. Six to eight-weeks old male C57BL/6 mice (20–23 g) were purchased from Beijing Vital River Company and housed in an environmentally controlled facility with free access to food and water. One hundred mice were randomly divided into four groups: sham, sham + fish oil, TBI and TBI + fish oil. Mice in the sham and TBI groups were on a regular diet containing a low concentration of Omega-3 PUFAs and mice in the sham + fish oil and TBI + fish oil were on the same diet supplemented with triple strength omega-3 fish oil (15 g/kg, eicosapentaenoic and docosahexaenoic acids, Puritan's Pride, Oakdale, NY) for 2 months prior to introduction of TBI. This dose was selected according to previous studies, which showed that pretreatment of mice with 15 g/kg fish oil for 2 months prior to TBI induction resulted in significant neuroprotection (10, 15). To reduce the oxidation of Omega-3 PUFAs, diets were vacuum packaged, stored at −20°C and allowed to reach room temperature before being served.

To confirm the enrichment of fatty acids in mice treated with fish oil, we assessed fatty acid profile in the brain before the induction of TBI through capillary gas chromatography as described previously (17) (Supplementary Figure 1). We found that both groups had similar levels of saturated fatty acids (SFA) and monounsaturated fatty acids (MUFA) and that mice treated with fish oil had significantly reduced Omega-6 PUFA and significantly increased Omega-3 PUFAs compared to that of the control group (Supplementary Figure 1a). The ratio of Omega-3/Omage-6 was significantly increased in mice treated with fish oil compared to that of the control group (Supplementary Figure 1b). Among the Omega-3 PUFAs, we found that fish oil treatment significantly increased the levels of docosahexaenoic acid (DHA), docosapentaenoic acid (DPA) and eicosapentaenoic acid (EPA), and not α-linolenic acid (ALA) (Supplementary Figure 1c). Among the Omega-6 PUFAs, we found that fish oil treatment significantly reduced the level of arachidonic acid (AA), increased the levels of γ-linoleic acid (GLA), and did not change the levels of docosatetraenoic acid (DTA) and dihomo-γ-linoleic acid (DGLA), compared to that of the control group (Supplementary Figure 1d).

TBI was induced mice in the TBI and TBI + fish oil groups by a controlled cortical impact (CCI) apparatus as described previously (18). Briefly, the mouse was anesthetized. The head was shaved and an incision was made to expose the skull. A hole of 3.5 mm in diameter was drilled on the right hemisphere 2.0 mm lateral to the midline and 2.0 mm from Bregma. TBI was induced with the impact applicator applied at a depth of 2 mm at 4.5 m/s for 200 ms. After application of the impact, the scalp was sutured. The mouse was recovered on a heating pad. One mouse died in anesthesia and two mice died after TBI surgery.

Mice in the sham and sham + fish oil groups underwent the same procedure without the cortical impact.

All animal studies were reviewed and approved by the Ethical Committee in Hebei Medical University.

### Neurological Functional Assessment by Modified Neurological Severity Score (mNSS)

The neurological functions of the mice were evaluated by mNSS prior to surgery and 1 day (d), 3 d, 5 d and 7 d after surgery. mNSS assesses the reflex, balance, sensory and motor functions in the mice with a point assigned to the mouse for abnormal behavior in each task so that the maximal mNSS was 18 indicating maximum neurological impairment and the minimal mNSS was 0 indicating absence of impairment (18).

### Rota-Rod Test

Motor coordination of the experimental mice was further determined by the Rota-rod test (18) on the day of surgery prior to craniotomy and at days 1, 3, 5, and 7 after the surgery. Briefly, each mouse was placed on an accelerating Rota-rod from 4 to 40 rpm for a total of 5 min. During each day, the mouse was tested for 4 trials with a 30 min interval between trials. The latency to fall was recorded. It the mouse stayed on the rod for the entire trial, 300 s was defined as the latency to fall. The average of latency to fall of all four trials was calculated for each mouse.

### Assessment of Lesion Volume

The volume of the lesion following TBI induction was assessed as described previously (10). Briefly, mice were transcardially perfused with 4% paraformaldehyde after anesthesia. Brains were extracted, incubated in 20% sucrose, embedded in OCT and sliced into 25 μm sections. Brain sections underwent hematoxylin and eosin (H&E) staining and were imaged. Lesion volume was measured using the ImageJ software.

### Enzyme-Linked Immunosorbent Assay (ELISA)

Aß42 level in the cerebral cortex was assessed as described previously (19). Briefly, at day 7 post CCI operation, a subset of mice was sacrificed under anesthesia (*n* = 6 for each group) and the cerebral cortex was isolated and frozen immediately. An equivalent amount of cerebral cortex from each mouse was homogenized, sonicated and centrifuged. The level of Aß42 in the supernatant was determined by an ELISA kit according to manufacturer's instruction.

### Radioisotope Clearance Assay

Radioisotope clearance assay was performed to determine the integrity of the glymphatic system according to the previous study (11). Briefly, on the 6th day after TBI induction, mice were anesthetized and a guide cannula was implanted into the frontal cortex of the contralateral hemisphere. One day after the implantation, 0.05 μCi of radiotracers including ^14^C-inulin and ^3^H-mannitol in 500 nl artificial CSF were infused into the parenchyma of the brain through the cannula for 5 min. The mouse was sacrificed at 1 h after the beginning of the infusion. The brain was isolated and solubilized overnight in 500 μl of tissue solubilized. Radioactivity was determined after adding 500 μl of liquid scintillation cocktail. Clearance of ^14^C-inulin and ^3^H-mannitol in 1 h was calculated by subtracting the ratio of radioactivity at 1 h from 100 percent.

### Western Blot Analysis

Mice were sacrificed under anesthesia at 7 days following TBI induction. The brain tissue adjacent to the site of the impact was isolated and lysed in RIPA buffer containing phosphatase and protease inhibitors. An equivalent amount of proteins was subjected to Western blot analysis by electrophoresis and incubation with respective antibodies as described previously (20). Primary antibodies used in this study included AQP4 (Santa Cruz Biotechnology, diluted at 1:500), ß-actin (Cell Signaling Technology, diluted at 1:1,000), ZO-1 (Cell Signaling Technology, diluted at 1:500), and Occludin (Cell Signaling Technology, diluted at 1:500).

### Quantitative Real-Time PCR (qRT-PCR)

qRT-PCR was performed to determine the mRNA expression of AQP4 as described previously (21). Briefly, the total RNA was isolated from ipsilateral hemispheres of the mouse brain extracted at 7 days after CCI using the Trizol reagent (Invitrogen). The Prime-Script RT reagent kit (Takara Bio.) was used for reverse transcription. Primers used in this study included: AQP4: 5′-CTGGAGCCAGCATGAATCCAG-3′ (forward), 5′- TTCTTCTCTTCTCCACGGTCA−3′ (reverse); GADPH: 5′-AGGTCGGTGTGAACGGATTTG-3′ (forward), 5′- TGTAGACCATGTAGTTGAGGTCA-3′ (reverse). GADPH was used as an internal control for the calculation of relative AQP4 expression.

### Immunofluorescent Staining

AQP4 polarization in the mouse brain was examined by immunofluorescent staining as described previously (15). Briefly, mouse was anesthetized and transcardially perfused with phosphate-buffered saline (PBS) and 4% paraformaldehyde (PFA). Brain was isolated, fixed in 4% PFA for overnight, dehydrated in 30% sucrose solution for overnight, embedded in OCT and cryosectioned into 20 μm slices. The frozen sections were then subjected to immunofluorescent staining by blocking in 10% normal donkey serum for 1 h at room temperature, incubation in primary antibodies at 4°C for overnight and secondary antibodies for 1 h at room temperature. Primary antibodies used in this study included anti-AQP4 and anti-GFAP. AQP4 was normally localized to the paravascular endfeet and was depolarized when it was localized to the astrocytic soma (parenchyma domains) (19). To measure AQP4 polarity, the area of the image with a pixel intensity greater than or equal to that of the perivascular endfeet was calculated (value expressed as a percentage of total field of view). Five randomly selected slices per animal were analyzed and ten randomly selected regions per slice were measured by individuals masked to the experimental group with ImageJ software. Data were normalized to the sham group.

### Evans Blue Extravasation

Evans blue extravasation was assessed to determine blood-brain barrier integrity as described previously (21). Briefly, on the 7th day post CCI, the Evans blue dye was administered to the mouse by intravenous injection. Two hours after injection, the mouse was perfused with PBS under anesthesia to remove intravascular dye. The brain was isolated and each hemisphere was homogenized with 1 mL 50% trichloroacetic acid solution. After centrifugation for 20 min at 12,000 × g, the supernatant from the homogenate was diluted with ethanol at a ratio of 1:3. The amount of Evans blue in the homogenate was determined by a spectrophotometer detecting absorbance at 610 nm and calculated according to a standard curve.

### Statistical Analysis

Data were represented by mean ± standard deviation (S.D). Differences between groups were determined by one or two-way ANOVA analysis with an appropriate *post hoc* test, and were regarded as statistically significant with *p* < 0.05.

## Results

### Omega-3 Attenuated TBI Induced Neurological Impairment

**Figure 1:**
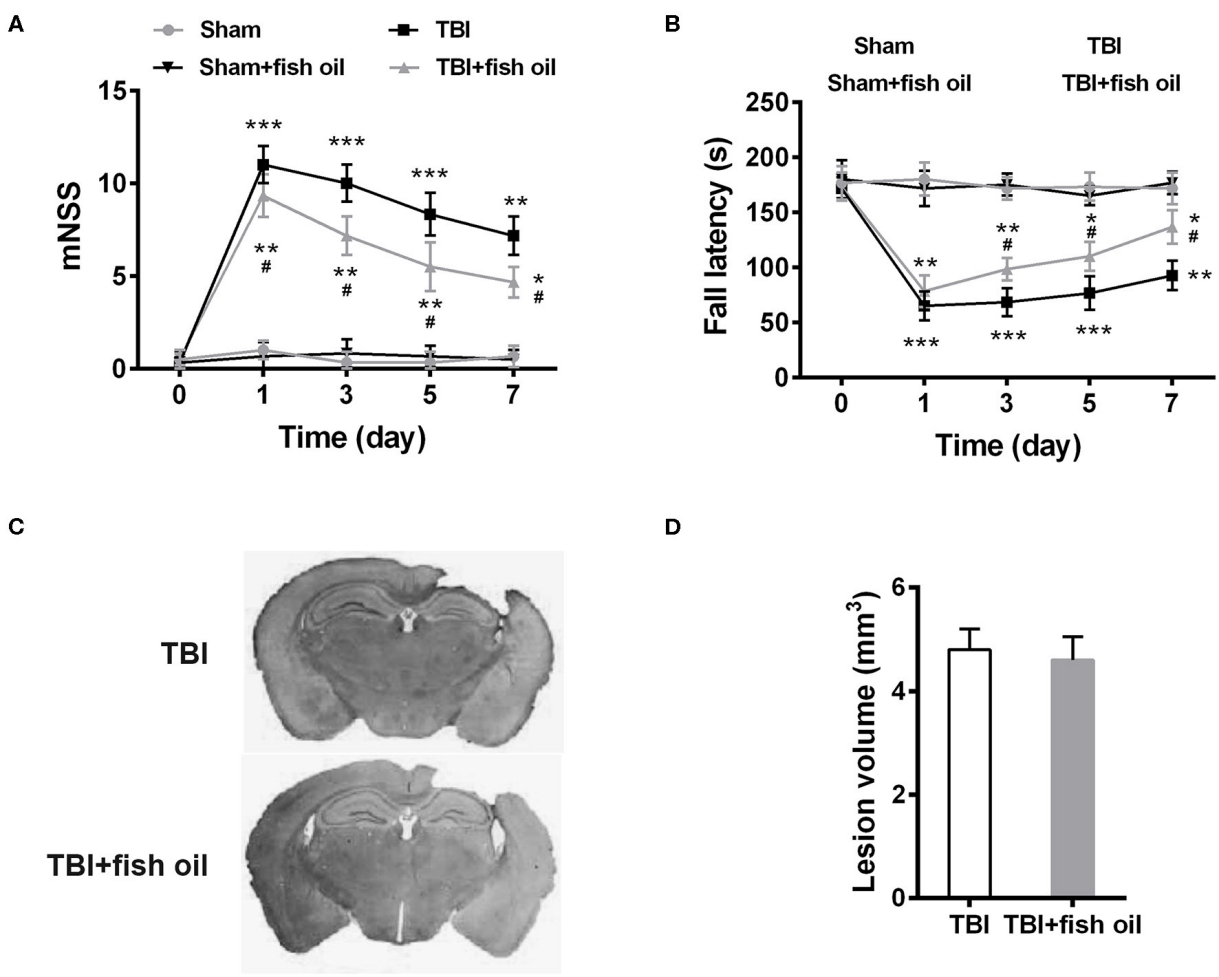
Omega-3 ameliorated neurological dysfunctions after TBI. The neurological recovery was analyzed by mNSS **(A)** and Rota-rod **(B)** tests prior to and at 1, 3, 5, 7 days post-TBI. **(C)** Brains were serially sliced and stained by H&E. **(D)** Lesion volume was quantified. Data are presented as mean ± SD. *n* = 10 for each group. *p < 0.05, **p < 0.01, ***p < 0.001 compared with sham group, #*p* < 0.05 compared with TBI group at the same time point. Statistical analysis was performed using two-way ANOVA followed by Bonferroni *post hoc* test.

We first assessed neurological function through behavioral assays. Mice in all four groups showed excellent neurological function prior to the TBI induction indicated by the low score of the mNSS assessment (Figure 1A). Sham operation or fish oil supplementation prior to the operation did not significantly increase mNSS, suggesting that sham operation only or together with fish oil supplementation did not induce neurological deficits. Expectedly, mice in the TBI group showed significant neurological impairment following CCI as indicated by increased mNSS assessed at days 1, 3, 5, and 7 after TBI induction. Importantly, fish oil supplementation for 2 months prior to TBI induction significantly reduced mNSS, although it was still higher than that of the sham operated mice.

We further assessed motor coordination by Rota-rod test. Consistent with the mNSS, Sham operated mice showed excellent motor coordination and fall latency did not change significantly from that prior to the operation (Figure 1B). Fish oil supplementation had no effect on sham operated mice. CCI significantly reduced fall latency and the impairment was present at 7 days' post operation. Similar to the mNSS, mice supplemented with fish oil showed significant improved performance in the Rota-rod test after induction of TBI.

Since no significant differences in the neurological functions were detected between the sham and sham + fish oil groups, we only used the sham group as control for the subsequent experiments.

To determine whether the differences in the neurological functions were due to differences in lesion size, we assessed lesion volume in mice of the TBI and TBI+fish oil groups (Figure 1C). We found that mice treated with fish oil did not differ significantly in the volume of the lesion after TBI induction compared to that of mice undergoing TBI alone (Figure 1D).

### Omega-3 Improved Glymphatic Clearance After Induction of TBI in Mice

**Figure 2:**
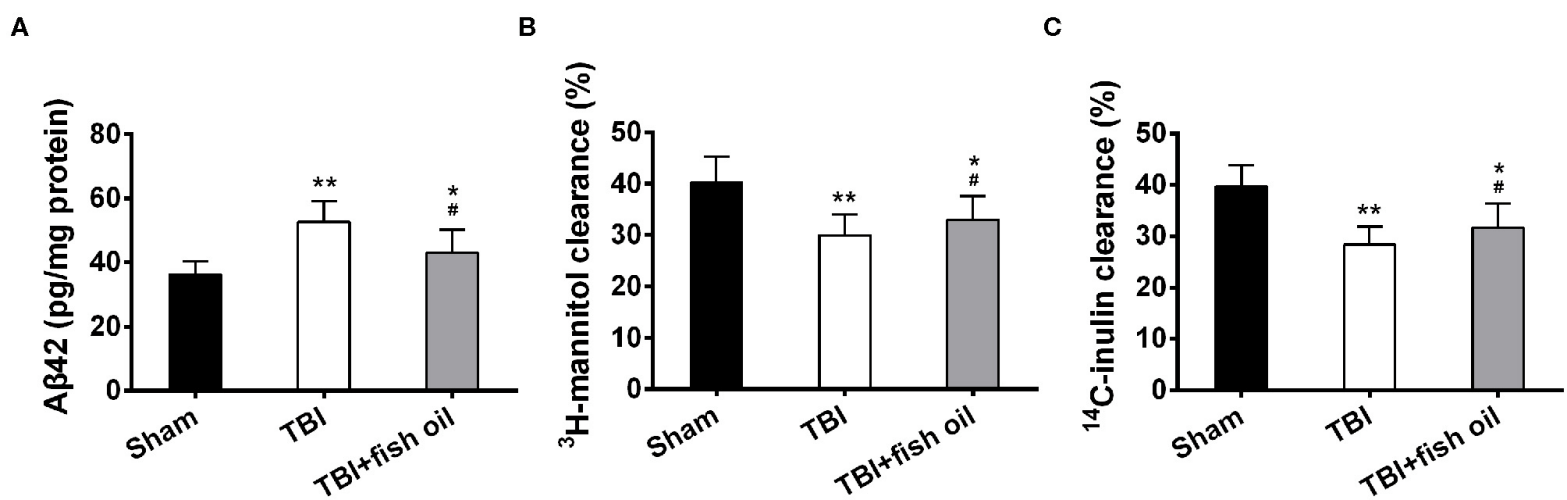
Omega-3 alleviated Aβ42 accumulation and improved glymphatic clearance after TBI. **(A)** Levels of soluble Aβ42 in the cerebral cortex were detected with ELISA. The clearance of radiolabeled ^3^H-mannitol **(B)** and ^14^C-inulin **(C)** was measured 60 min after infusion into frontal cortex. Data are presented as mean ± SD. *n* = 6 for each group. *p < 0.05, **p < 0.01 compared with sham group. #*p* < 0.05 compared with TBI group. Statistical analysis was performed using one-way ANOVA followed by Bonferroni *post hoc* test.

Previously, it has been shown that TBI induces Aß42 accumulation and that glymphatic system is involved in the clearance of Aß42 (19). We assessed the effect of fish oil pretreatment on TBI induced Aß42 production in the mouse cortex. We found that TBI significantly increased the level of Aß42 in mouse cortex compared to that in sham mice, and that fish oil supplementation significantly reduced Aß42 accumulation (Figure 2A). Our study suggested that mice pretreated with fish oil might have improved Aß42 clearance. However, we could not exclude that possibility that fish oil supplementation might suppress TBI induced excessive Aß42 production. To further determine the function of glymphatic system, we measured radioisotope clearance and found that the clearance of both ^3^H-mannitol (Figure 2B) and ^14^C-Inulin (Figure 2C) was significantly reduced in mice with TBI, and supplementation of the mice with fish oil for 2 months prior to CCI partially restored the clearance of both radioisotopes.

### Omega-3 Suppressed AQP4 Expression Following TBI

**Figure 3:**
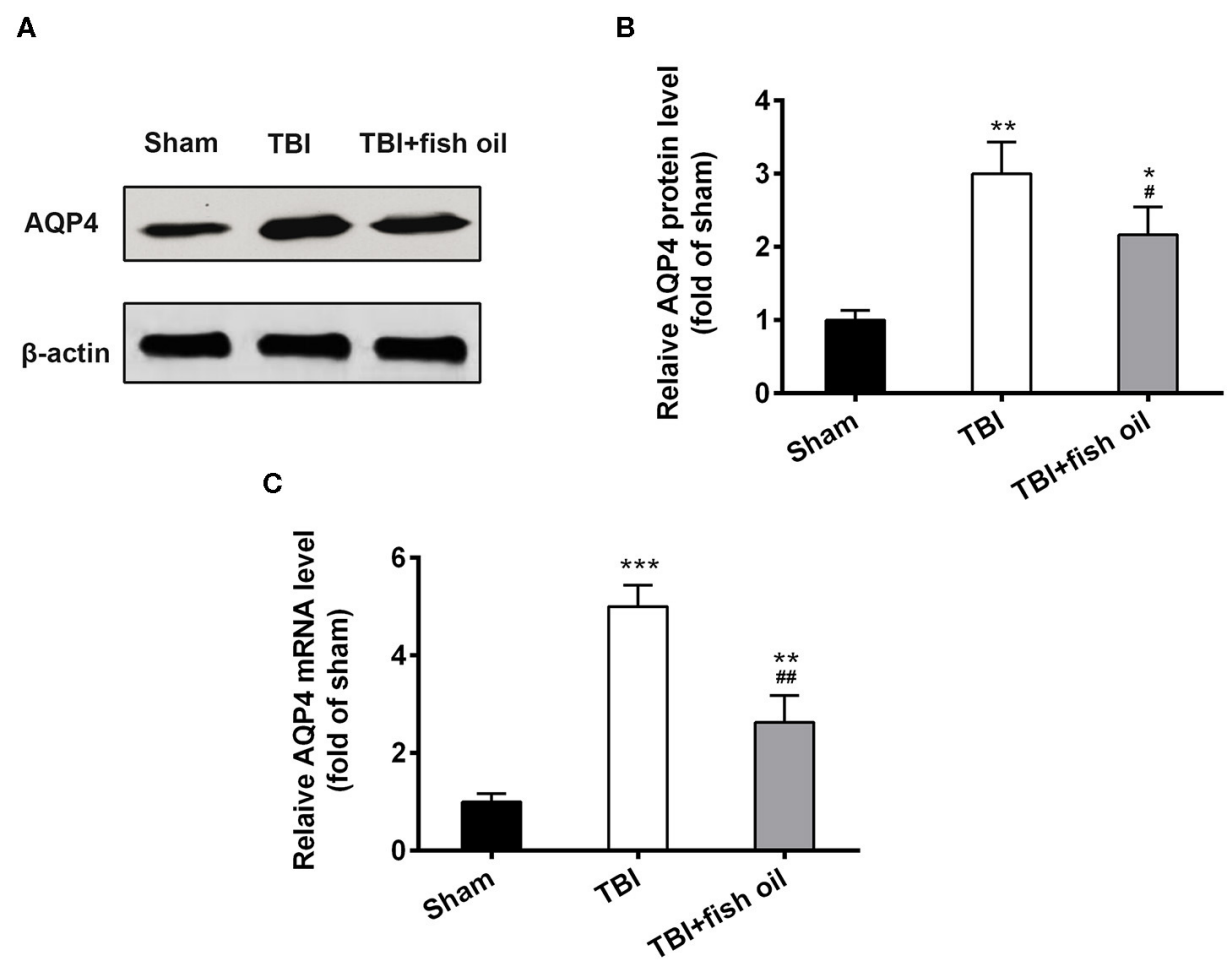
Omega-3 reduced the expression of AQP4 after TBI. **(A)** Western blot showed the significant increase in AQP4 protein expression following TBI and Omega-3 treatment reduced AQP4 expression. **(B)** Relative AQP4 protein level from western blot was analyzed. **(C)** Relative AQP4 mRNA level was detected by qRT-PCR. Data are presented as mean ± SD. *n* = 6 for each group. *p < 0.05, **p < 0.01, ***p < 0.001 compared with sham group. #*p* < 0.05, ##*p* < 0.01 compared with TBI group. Statistical analysis was performed using one-way ANOVA followed by Bonferroni *post hoc* test.

AQP4 is an important protein involved in the glymphatic system. Western blot analysis showed that AQP4 protein expression was significantly increased in the brain of mice with TBI compared to that of sham operated mice (Figures 3A,B), which was significantly suppressed by pretreatment of the mice with fish oil. Consistently, the mRNA level of AQP4 was also drastically elevated in the brain of mice with TBI and fish oil supplementation significantly reduced AQP4 mRNA expression in mice undergoing CCI (Figure 3C).

### Omega-3 Partially Prevented Loss of AQP4 Polarity in Mice With TBI

**Figure 4:**
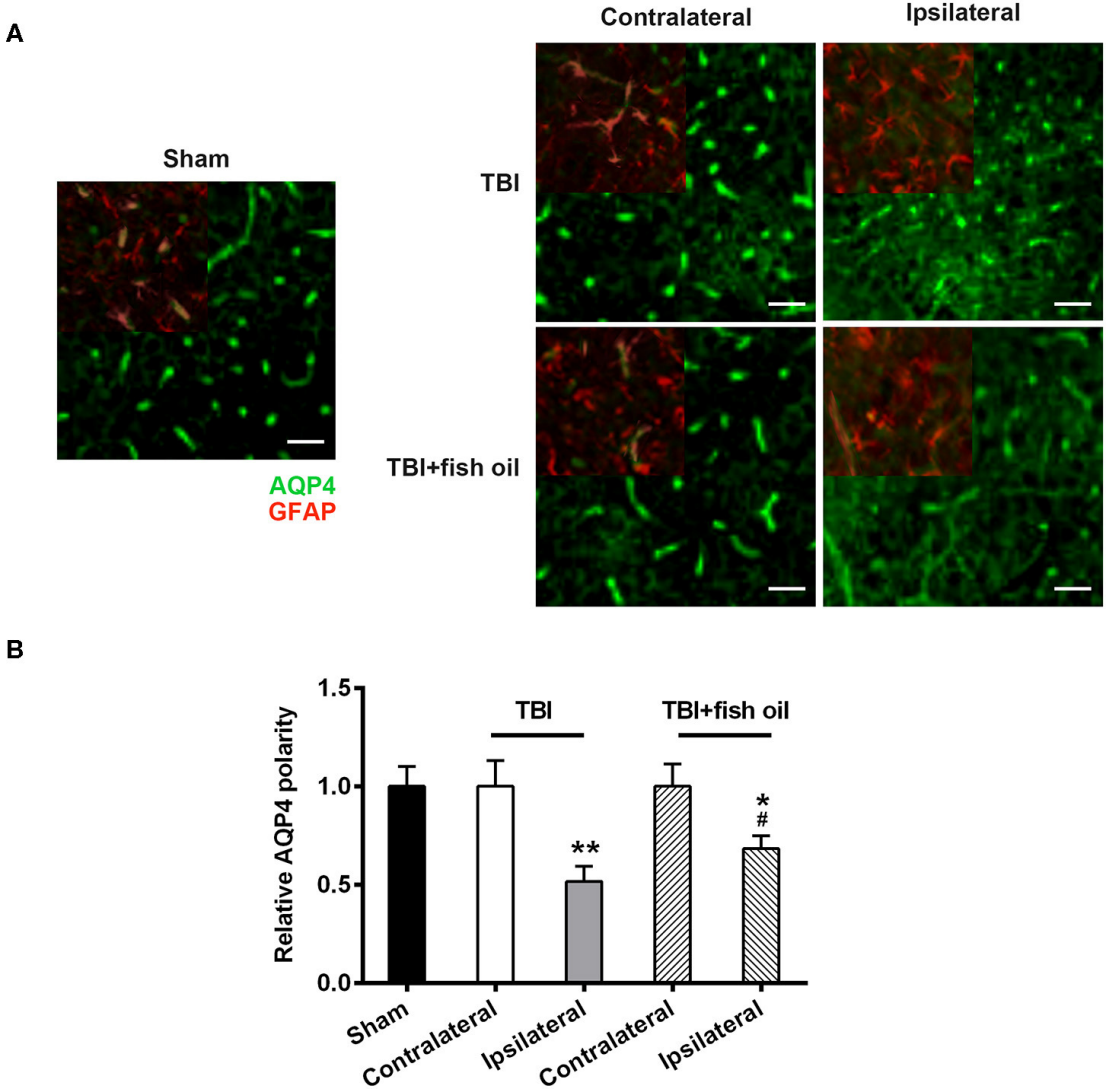
Omega-3 ameliorated disruption of AQP4 polarity after TBI. Representative images **(A)** and quantification **(B)** of AQP4 polarization in different groups 7 days post-TBI. Data are presented as mean ± SD. n = 6 for each group. *p < 0.05, **p < 0.01 compared with sham group. #*p* < 0.05 compared with TBI group. Statistical analysis was performed using one-way ANOVA followed by Bonferroni *post hoc* test. Scale bars: 25 μm.

We examined the polarity of AQP4 by staining the brain with both AQP4 and GFAP immunofluorescence staining (Figure 4A). We found that AQP4 polarity was significantly disrupted on the ipsilateral cortex in mice with TBI compared to that of the sham group (Figure 4B) which was partially and significantly restored by pretreating the mice with fish oil. AQP4 polarity on the contralateral side was not significantly affected by CCI operation.

### Omega-3 Partially Prevented TBI Induced Blood-Brain Barrier Disruption

**Figure 5:**
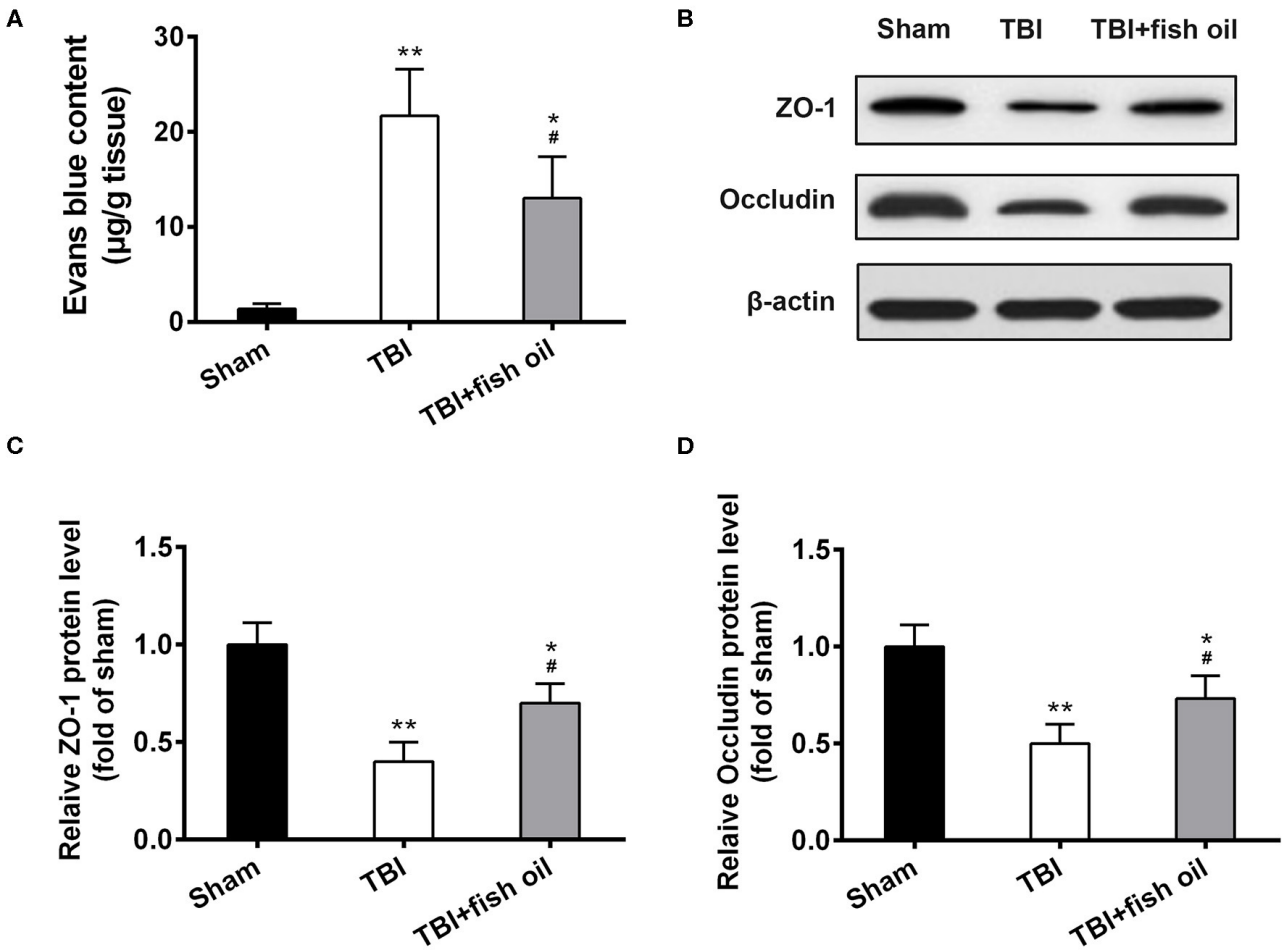
Omega-3 protected the integrity of the blood-brain barrier after TBI. **(A)** Evans blue content in the brain was quantified. ZO-1 and occludin protein expression was detected by western blot **(B)** and relative protein levels of ZO-1 **(C)** and occludin **(D)** from western blot were analyzed 7 days post-TBI. Data are presented as mean ± SD. *n* = 6 for each group. *p < 0.05, **p < 0.01 compared with sham group. ^#^*p* < 0.05 compared with TBI group. Statistical analysis was performed using one-way ANOVA followed by Bonferroni *post hoc* test.

Finally, we investigated the effect of Omega-3 in the integrity of blood-brain barrier. Analysis of Evans blue content in the brain homogenate showed that TBI significantly increased Evans blue extravasation (Figure 5A) compared to sham operated mice. Pretreatment of the mice with fish oil prior to CCI operation significantly alleviated blood-brain barrier disruption by TBI.

We further assessed the protective effect of fish oil on blood-brain barrier structure by examination of ZO-1 and occluding proteins using Western blot analysis (Figure 5B). We found that TBI induced significant loss of both ZO-1 and occluding in the mouse brain, and importantly, fish oil significantly although partially prevented lZO-1 and occluding loss caused by CCI operation (Figures 5C,D).

## Discussion

In this study, we investigated the role of Omega-3 by fish oil supplementation on the neurological functions in a mouse model of TBI and explored the underlying structural and molecular mechanisms. We confirmed that TBI induced significant impairment in neurological functions as depicted by substantial increase in the mNSS scores and disrupted motor learning and coordination in the Rota-rod test. We found that 2 months' treatment of fish oil in mice prior to TBI induction by CCI significantly alleviated neurological impairment in both behavioral assays while fish oil treatment in sham operated mice had no obvious beneficial or devastating effects on the neurological function. We confirmed that TBI induced significant deficits in the glymphatic system resulting in substantial accumulation of Aß in the brain and poor clearance of radiolabeled tracers. Our study showed that the waste clearance function of the glymphatic pathway was significantly rescued by Omega-3 which not only partially prevented accumulation of the neurotoxic Aß but also significantly promoted clearance of radiolabeled tracers in the brain following TBI induction. Our found that AQP4 might be involved in the regulation of the glymphatic pathway by Omega-3. Both the expression and polarity of AQP4 were significantly disrupted by TBI which were partially restored by Omega-3. Finally, our study showed that Omega-3 played a protective role in maintaining the integrity of blood-brain barrier. TBI induced extravasation of Evans blue was significantly restored in mice pretreated with fish oil. Additionally, Omega-3 also restored the protein levels of two main components of the tight junctions of the blood brain barrier, occludin and ZO-1 which were suppressed by TBI.

Disrupted glymphatic system has been shown to be associated with TBI (11). Previous studies show that mice undergoing CCI operation have impairment clearance of interstitial solute, leading to accumulation of neurotoxic substances such as aggregated Tau (11). Fish oil administration in mice prior to Aß injection protects mice against Aß induced neurotoxicity and promotes Aß clearance (15). We thus hypothesized that long term treatment of the mice with Omega-3 might prevent TBI induced disruption of the glymphatic system. In our study, we observed a consistent impairment in glymphatic clearance in mice following TBI induction. Not only more Aß42 was accumulated but clearance of radiolabeled tracers was also significantly hindered in mice after TBI induction. Supporting our hypothesis, our study showed that Omega-3 administration prior to TBI induction significantly suppressed Aß42 accumulation and promoted clearance of radiolabeled tracers. Our study together with others suggest that long term treatment of mice with Omega-3 partially restores waste clearance mediated by the glymphatic system in mice undergoing CCI surgery.

AQP4 is an important protein involved in the glymphatic system. Deletion of AQP4 in mice abolished Omega-3 induced restoration of glymphatic function following TBI induction indicated by exacerbated clearance of radiolabeled tracers from the brain (15). In a mouse model of chronic stress, glymphatic transport system is disrupted which is accompanied by reduction of AQP4 protein and mRNA expression and inhibition of AQP4 polarization (19). Aß plaque accumulation is increased in the absence of AQP4 in a mouse model of Alzheimer's disease (22). Similarly, another study also showed that mice lacking AQP4 had reduced Aß42 clearance (6). These studies suggest that AQP4 is required for Aß42 clearance. On the other hand, our study showed that increased AQP4 expression as a result of TBI was accompanied with Aß42 accumulation. Our study together with the previous studies suggest that a certain level of AQP4 is required for optimal clearance of Aß42, with both too much and too little AQP4 resulting in impaired Aß42 clearance.

Importantly, our study further showed that administration of the mice with Omega-3 significantly reversed AQP4 expression and suppressed AQP4 depolarization in the TBI mice. Since AQP4 polarization in the astroglial cells is important for the formation of the glymphatic tunnels, our study suggests that long term administration of Omega-3 in mice protects the glymphatic system from TBI induced disruption by maintaining astroglial function.

In addition to the glymphatic pathway, the blood-brain barrier is also a critical component in the maintenance of central nervous system homeostasis. The blood-brain barrier not only restricts substance entrance to the brain from the blood by passive diffusion, but also facilitates active nutrient transport to the brain and efflux of toxic metabolites out of the brain (23). Breakdown of the blood-brain barrier is often found in patients following TBI (24, 25). Our study of the Evans blue extravasation assay confirmed the disruption of blood-brain barrier by TBI. Consistent with previous studies (20), we found that TBI lead to accumulation of Evans blue in the brain, an indication of undermined integrity of the blood-brain barrier and importantly, our study showed that this disruption was partially restored by Omega-3 pretreatment. Furthermore, we found that TBI led to suppressed expression of ZO-1 and occludin, two major components of the tight junctions that are important for blocking passive diffusion and active transportation of substances in and out of the brain (26). Our study showed that ZO-1 and occludin protein expression levels were significantly restored in mice supplemented with fish oil in the diet prior to the induction of TBI.

As important structural components of the neuronal membranes, members of the Omega-3 series have been shown to be essential for brain function and sufficient Omega-3 intake is required for neural development (27). High intake of Omega-3 is associated with a reduced risk of dementia in aging population (28). Consistently, our study along with others have identified striking beneficial effect of long term Omega-3 intake in the prevention of TBI induced neurological impairment without affecting the normal function of sham operation mice. Although beyond the scope of this study, one future direction may be that if Omega-3 treatment following TBI has any beneficial effect in restoring neurological function.

## Conclusion

In summary, our study reveals important molecular mechanisms by which Omega-3 alleviates TBI induced impairment in neurological functions. We show here that Omega-3 protects the function of glymphatic system by maintaining the level and polarity of AQP4 and regulates the integrity of blood-brain barrier by suppressing loss of tight junction components induced by TBI. Evidence in our study thus suggests that nutritional Omega-3 supplementation has a neuroprotective effect against TBI through maintenance of waste clearance and substance exchange in and out of the brain.

## Data Availability Statement

The raw data supporting the conclusions of this article will be made available by the authors, without undue reservation, to any qualified researcher.

## Ethics Statement

The animal study was reviewed and approved by Ethical Committee in Hebei Medical University.

## Author Contributions

ZF and LY designed research. EZ, DW, XW, YC, ZC, ML, GZ, JW, and HH performed research. ZF and LY analyzed data. LY and EZ wrote the paper. All authors contributed to the article and approved the submitted version.

## Conflict of Interest

The authors declare that the research was conducted in the absence of any commercial or financial relationships that could be construed as a potential conflict of interest.
