# Supplementary material for: Omega-3 Polyunsaturated Fatty Acids Alleviate Traumatic Brain Injury by Regulating the Glymphatic Pathway in Mice
Source: Front Neurol. 2020 Jul 17;11:707. doi: 10.3389/fneur.2020.00707 (PMC7380115; doi:10.3389/fneur.2020.00707)
Supplement: Supplementary file 1 [file Data_Sheet_1.PDF]

Supplementary Materials

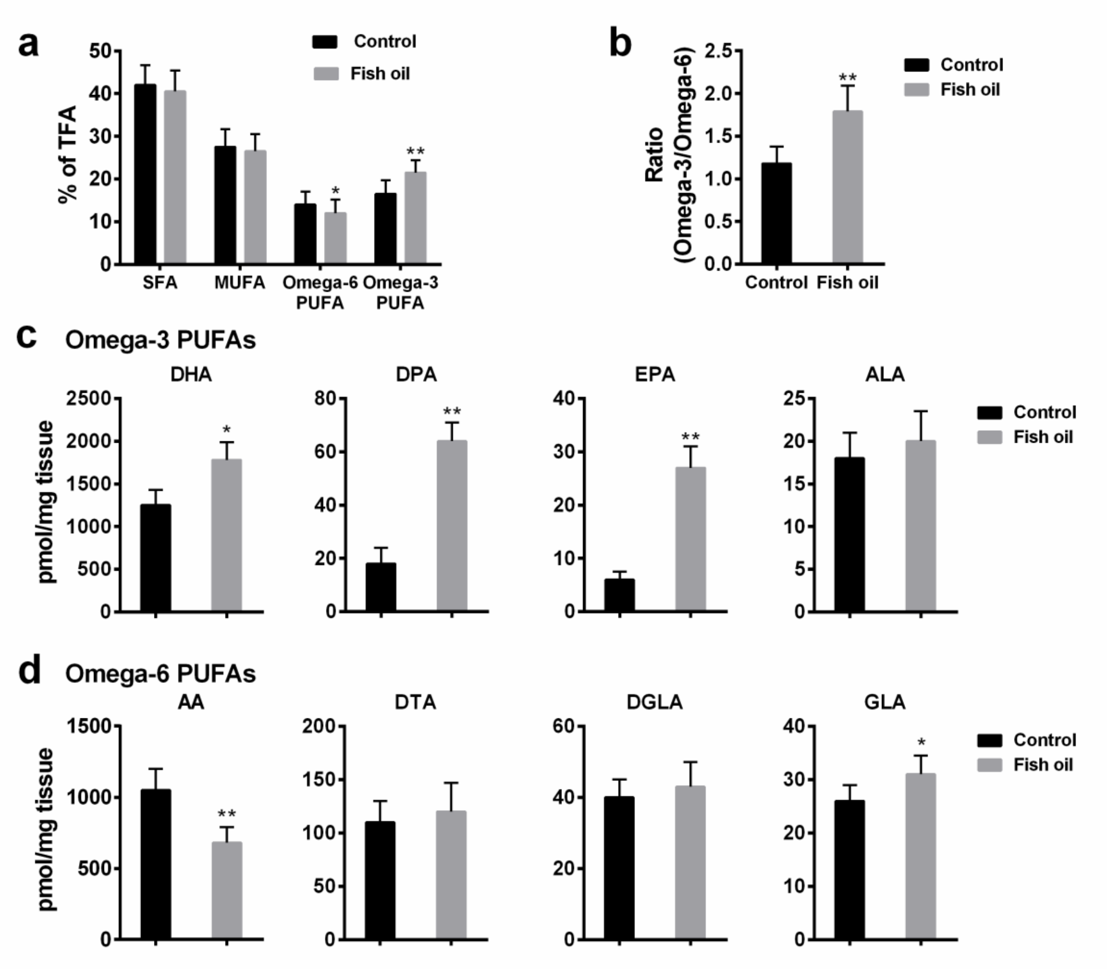

Supplementary Figure S1. Lipid profiles in the brain of the mice on diet supplemented with triple strength omega-3 fish oil and on regular diets. (a) Lipid profiles in mouse brains expressed as the percent of total fatty acids (TFA). (b) The omega-3/omega-6 ratio increased in mice fed with omega-3 enriched diets compared to those on a regular diet. Specific omega-3 PUFAs (c) and omega-6 PUFAs (d) content. Data are presented as mean ± SD. n = 3 for each group. *p < 0.05, **p < 0.01 compared with control group.
